# The SENIEUR protocol and the efficacy of hepatitis B vaccination in healthy elderly persons by age, gender, and vaccine route

**DOI:** 10.1186/s12979-020-00179-9

**Published:** 2020-04-28

**Authors:** Robert Edelman, Meagan E. Deming, Franklin R. Toapanta, Mark D. Heuser, Lisa Chrisley, Robin S. Barnes, Steven S. Wasserman, William C. Blackwelder, Barry S. Handwerger, Marcela Pasetti, Khan M. Siddiqui, Marcelo B. Sztein

**Affiliations:** 1 Center for Vaccine Development and Global Health, University of Maryland School of Medicine, Baltimore, USA; 2 Department of Medicine, University of Maryland School of Medicine, Baltimore, USA; 3 Institute of Human Virology, University of Maryland School of Medicine, Baltimore, USA; 4 Present Adress: Department of Veterans Affairs, Salisbury VA Health Care System, Salisbury, NC USA; 5 Present Adress: Office of Research, University of Virginia, Charlottesville, USA; 6 Department of Epidemiology and Public Health, University of Maryland School of Medicine, Baltimore, USA; 7 Rheumatology and Clinical Immunology, Dept of Medicine, University of Maryland School of Medicine, Baltimore, USA; 8 Department of Pediatrics, University of Maryland School of Medicine, Baltimore, USA; 9 Department of Microbiology and Immunology, Dept of Medicine, University of Maryland School of Medicine, Baltimore, USA; 10 Imaging Informatics and Body Magnetic Resonance Imaging unit, Veterans Affairs Maryland Health Care System Baltimore, Baltimore, MD USA; 11 Present Adress: Russell H. Morgan Department of Radiology and Radiological Science, Johns Hopkins University School of Medicine, Baltimore, USA

**Keywords:** Hepatitis B, Vaccine, Aging, Immunesenescence, Antibody, Cellular immunity

## Abstract

**Background:**

Reduced response to hepatitis B vaccines is associated with aging, confounding and comorbid conditions, as well as inadvertent subcutaneous (SC) inoculation. We hypothesized that the antibody and T cell-mediated immune responses (T-CMI) of elderly adults to a vaccine intended for intramuscular (IM) administration would be attenuated when deposited into SC fat, independent of confounding conditions.

**Results:**

Fifty-two healthy, community dwelling elderly adults (65–82 years), seronegative for HBV, were enrolled in the SENIEUR protocol as a strictly healthy population. These seniors were randomized to receive a licensed alum-adjuvanted recombinant HBV vaccine either SC or IM, with the inoculum site verified by imaging. The response rates, defined as hepatitis B surface antibodies (HBsAb) ≥10 IU/L, were significantly lower in the elderly than in young adults, a group of 12, healthy, 21–34-year-old volunteers. Moreover, elderly participants who received the vaccine IM were significantly more likely to be responders than those immunized SC (54% versus 16%, *p* = 0.008). The low seroconversion rate in the IM group progressively declined with increasing age, and responders had significantly lower HBsAb titers and limited isotype responses. Moreover, T-CMI (proliferation and cytokine production) were significantly reduced in both percentage of responders and intensity of the response for both Th1 and Th2 subsets in the elderly.

**Conclusions:**

Our data demonstrate the blunted immunogenicity of SC inoculation as measured by peak titers and response rates. Further, the qualitative and quantitative deficits in B- and T-CMI responses to primary alum adjuvanted protein antigens persisted even in strictly healthy elderly populations with verified IM placement compared to younger populations.

**Clinical trial registration:**

ClinicalTrials.gov, NCT04162223. Registered 14 November 2019. Retrospectively registered.

## Background

Vaccines remain a powerful tool for the prevention of infectious diseases in the elderly, but older adults respond less vigorously to vaccinations (e.g., influenza, pneumococcal pneumonia, zoster, and Hepatitis B) when compared to younger individuals [1, 2]. This phenomenon is ascribed to age-related alterations of the immune system, or immunesenescence, which is caused by multiple factors including deficits in T cell proliferation, signaling, and activation, in T cell help to B cells, in expression of Toll-like receptors (TLRs) and antigen presentation, as well as waning of naïve B and T cell populations [3–7]. Most vaccinations recommended for elderly populations include antigens for which there is already immunological memory (e.g., seasonal influenza virus, Tdap, shingles vaccine) [2, 8, 9]. In contrast, primary immunizations against HBV are recommended for high risk groups including patients requiring dialysis, receiving immunosuppression therapy, those affected with chronic liver disease, HIV, HCV, or travelers [10]. Reduced rates of protection (Ab responses ≥10 IU/ml) have been reported in volunteers > 60 years of age receiving alum-adjuvanted HBV vaccines, ranging from 34% in the frail elderly to 72% in clinical trial populations [11–13]. Despite the lower protection rates following primary vaccination for HBV, booster responses for older adults (> 65 years) after re-vaccination (for individuals with documented vaccination > 10 years prior) appear to be intact in elderly, suggesting that the ability of T cells to generate lasting memory following a di novo vaccination declines with age [14].

The intrinsic changes due to immunesenescence are confounded by several factors modulating immune responses. Several host attributes associated with diminished responses to HBV vaccination include comorbidities (HIV, end-stage renal disease [ESRD], obesity), health habits (smoking and alcohol abuse), and male gender [15–19]. Moreover, the depressive effects of protein energy malnutrition on immune function have been well described, and linked in particular to impaired responses to HBV vaccination [20]. Low vitamin E (tocopherol) levels are associated with reduced antibody titers responses to HBV vaccination [21]. Reduced zinc levels are associated with decreased responsiveness to diphtheria and influenza vaccination, and similarly reduced responses to pneumococcal polysaccharide vaccination has been observed with reduced serum vitamin B_12_ levels [22–24]_._ Of note, the deltoid muscle is the preferred site of immunization in the elderly receiving influenza, pneumococcal, tetanus, and HBV vaccines. However, aging is associated with thickening of the fat pad overlying the deltoid muscle, which may result in the inadvertent delivery of vaccine antigens into subcutaneous fat using the standard hypodermic needles routinely supplied with single dose syringes [17, 25]. Therefore, injections intended for IM administration may be commonly deposited into the fat tissue of the elderly [26, 27]. Adipose tissue, with its relative dearth of immunologically active cells (e.g., lymphocytes, macrophages and dendritic cells) and reduced blood supply compared to muscle and derma, may contribute to the diminished primary immune response to vaccines observed in older adults. The decreased response, thus, may be due at least in part to the failure of immunogen and immune tissue interaction [15, 17, 27, 28]. The hydrophobic milieu of adipose tissue may further dampen immune responses by impeding the diffusion of cytokines and vaccine inoculum to immunologically active tissues [17].

As life expectancy continues to increase worldwide, increasing numbers of elderly individuals require vaccinations for which there is often reduced efficacy. Recombivax-HB (Merck and Co., Inc., West Point, PA), a licensed, alum-adsorbed recombinant HBV Surface Antigen (rHBsAg) protein vaccine was selected as a model vaccine and immunologic probe because it is safe, immunogenic and has been shown to have attenuated immune responses in diverse elderly populations. The purpose of this study was to measure the hepatitis B specific immune response in a strictly healthy elderly population receiving confirmed IM or SC injections, testing the hypothesis that the antibody response of older adults to a vaccine intended for IM administration will be attenuated with subcutaneous deposition. We further hypothesized that an otherwise strictly healthy elderly population would have persistent deficits in humoral and T cell-mediated responses (T-CMI) to vaccination even after verified appropriate IM administration.

## Results

### Baseline characteristics of the senior cohort

From the 613 persons initially contacted, 193 (31.4%) were excluded due to lack of interest in completing all study visits or by the lack of transportation to the testing site. Of the remaining 420 volunteers interested and available for the study, 368 (87.6%) were excluded based on strict inclusion and exclusion criteria (Table 1); 52 (12.4%) were enrolled and completed the study. The leading causes of exclusion were currently smoking ≥10 cigarettes daily or having a history of malignancy diagnosed or treated actively during the preceding 5 years.

**Table 1:** Inclusion and Exclusion Criteria

**Inclusion**	
1. Men and women 65 years of age or older	
2. Community-dwelling	
3. Normal range of reference laboratory for: complete blood count and differential, thyroid stimulating hormone, serum vitamin B_12,_ folate, vitamin E, AST/SGOT and ALT/SGPT, albumin, fasting blood glucose, blood urea nitrogen and serum creatinine	
4. Negative serum tests for hepatitis B and hepatitis C	
**Exclusion**	
1. History or clinically apparent immunologically mediated chronic conditions (e.g., rheumatoid arthritis, lupus erythematosus). No exclusion for stable chronic non-immunologically mediated conditions (e.g., osteoarthritis, well controlled hypertension)	
2. Immunodeficiency	
3. Severe respiratory disease requiring supplemental oxygen	
4. Psychiatric disorder, untreated or not in remission	
5. Infection within 2 weeks of immunization	
6. Inflammatory processes such as known chronic infections, inflammatory bowel disease or Westergren sedimentation rate (> 50 mm/hour for men, > 60 mm/hour for women)	
7. All malignancies (excluding non-melanotic skin cancer) and lymphoproliferative disorders diagnosed or treated actively during the past 5 years	
8. Arteriosclerotic event during the 2 weeks prior to enrollment (e.g., medically documented myocardial infarction, stroke, recanalization of the femoral arteries, claudication, or transient ischemic attack)	
9. Cardiac insufficiency, if heart failure present (New York Heart Association functional class III or IV)	
10. Poorly controlled hypertension (SBP ≥180 mmHg, DPB ≥100 mmHg)	
11. Renal Insufficiency (serum creatinine ≥2.0 or BUN ≥40)	
12. Elevated or low glucose (fasting ≥140 or < 70; non-fasting > 200)	
13. Cognitive impairment: score of < 23 on the Folstein Mini-Mental State Examination.	
14. Depression or mood alteration: score of ≥6 on the Geriatric Depression Scale	
15. Malnutrition as defined by clinical judgment and by decreased serum albumin (< 3.2 g/L) or hypocholesterolemia (< 160 mg/dL), or low total lymphocyte count (< 1500/ml^3^).	
16. Anemia (Hct < 30%) or low serum vitamin B_12,_ folate_,_ or vitamin E level	
17. History of or current alcoholism or consuming > 1 can of beer, 1 glass wine, or 1 oz. liqueur daily; current drug abuse; currently smoking ≥10 cigarettes per day.	
18. Risk factors for hepatitis B (such as parenteral drug abuse, multiple sexual partners, commercial sex worker, health care worker)	
19. History of hepatitis B infection or vaccination.	
20. Positive test for hepatitis B surface antigen or antibody, hepatitis B core antibody, or hepatitis C antibody.	
21. Unable to attend the Baltimore VA Medical Center on a regular basis; no telephone in primary residence.	
22. Subcutaneous fat pad less than 6 mm in thickness as determined by computer tomography	
23. Medication exclusions include prednisone > 5 mg/day (or equal), colchicine, methotrexate, azathioprine, cyclophosphamide, cyclosporine, or interferon.	

The cohort was robust, healthy and educated with 87% completing high school and 40% having at least 1 year of college education (Table 2). They performed basic and instrumental activities of living independently, with only one subject intermittently using a cane for ambulation. The median number of co-morbid conditions was three from a total of 12 possible co-morbid conditions, with a range of 0 to 7 conditions per volunteer. All volunteers screened negative for depressed mood and cognitive impairment. Ninety-six percent of the study cohort received influenza or pneumococcal vaccine in the year preceding the study. Four (8%) persons smoked less than 10 cigarettes per day, and 48 (92%) were non-smokers. Only 2 (4%) volunteers admitted to any alcohol consumption but no more than 1 can of beer, 1 glass of wine, or 1 oz. of liqueur daily, except for an individual who acknowledged drinking 2 cans of beer a day.

**Table 2:** Baseline Volunteer Characteristics for Senior and Junior cohorts

Characteristic	SENIOR IM	SENIOR SC	JUNIOR
Number (Percent) Male	13 (50%)	11 (42%)	6 (42%)
Number (Percent) White	25 (96%)	24 (92%)	7 (50%)
Number (Percent) African-American	1 (4%)	2 (8%)	4 (26%)
Median age [range]	73 [65–81]	72 [65–80]	26 [21–34]
Age 65–74 (percent)	20 (77%)	19 (73%)	n/a
Age 75–81 (percent)	6 (23%)	7 (27%)	n/a
Smoker (< 10 cigarettes per day)	0	0	1 (7%)
Median years of education [range]	12 [4–16]	12 [10–18]	16 [14–18]
Alcohol (> 1 can beer, 1 glass wine, or 1 oz. liqueur daily).	0	0	0
Previous influenza or pneumococcal immunization	25 (96%)	25 (96%)	0
Median number of medications [range]	4 [0–8]	3 [0–9]	1 [0–4]
Use of supplemental vitamins	18 (69%)	17 (65%)	5 (36%)
Median co-morbid conditions [range]*	3 [0–7]	3 [0–7]	Not done
Median weight (kg) [range]	79.5 [55–110]	79.5 [53–114]	
Median Body Mass Index [range]**	27.7 [22.3–38.6]	29.0 [20.0–41.9]	
Median body percentage fat [range]	37.1 [24.5–49.1]	36.9 [20.9–51.4]	
Median Charlson Index [range] ***	0 [0–3]	0 [0–3]	
Median Mini-Mental Status Exam score^+^	29 [23–30]	30 [25–30]	
Median OARS score [range]^#^	0 [0–2]	0 [0–0]	
Geriatric Depression Scale scores^##^	0 [0–2]	0 [0–2]	

Seniors were randomized to receive the Hepatitis B vaccine by intramuscular (IM) or subcutaneous (SC) administration. Baseline characteristics were similar between Senior IM and SC administration groups. (*) Based on 12 common medical conditions; none = 0, maximum =12. (**) BMI “Normal” =18.5–24.9; “overweight” = 25.1–29.9; “obese” grade 1 = 30–34.9, grade 2 = 35–39.9; grade 3 ≥ 40 [29]. (***) Prognostic 10-year survival for individuals with multiple comorbidities: 0 = 99%; 2 = 90% [30]. (+) Measure of cognitive impairment. Score 24–30 indicates no cognitive impairment [31]. (**#**) Older Americans Resources and Services, a measure of functional status. Score < 3 = no assistance required for daily activities [32]. **(**##**)** Any one symptom of depression; maximum depression score = 15 [33]**.** n/a = not applicable

The cohort was well nourished, as determined by body mass index (BMI), DEXA and chemistry parameters. Seventeen (32.7%) of volunteers were classified as obese by BMI (≥30); one was classified as morbidly obese (BMI of 42). The percentage total body fat by DEXA scan was consistent with the BMI measurements. Serum albumin (3.2–5.2 g/dL) and total lymphocyte counts were normal in all persons. Only 3 (6%) volunteers had cholesterol values < 160, but 18 (35%) had total cholesterol ≥ 220. The median, age matched, percent bone mineral density by DEXA scan was 105%, with a lowest value of 89%.

The clinical laboratory values were largely within the normal range. Only one person had a creatinine > 1.4 mg/dL, and 9 participants (17%) had a BUN > 20 mg/dL (21–26 mg/dL). Two persons had a low TSH (< 0.49 mc IU/ml), but they were clinically euthyroid; no one had clinical or laboratory evidence of hypothyroidism. The ALT values were normal (0–40 u/ml) in 51 of 52 persons (one person had 42 u/ml) and AST values were normal (0–37 u/ml) in 51 of 52 persons (one person had 51 u/ml). Serum fasting blood glucose level was normal in all persons. There was no clinical or laboratory evidence of anemia, except in one person with an Hgb of 11.9 and an Hct of 34%. The blood levels of folate, Vitamin B12, and Vitamin E, were within normal limits. Two-thirds of the cohort took vitamin supplements.

### Results of randomization

There were no statistically significant differences among the baseline characteristics of Senior volunteers randomized to the IM and SC treatment groups, including gender, age, smoking and drinking history, education level, body fat content, comorbid conditions, use of supplemental vitamins and vitamin levels, Mini-Mental Status exam, activity of daily living scale, and geriatric depression scale (Table 2).

### Baseline characteristics of the junior cohort

As a comparator to the responses observed in the elderly we included a cohort of young adults. All volunteers in this group were healthy travelers or students, Hepatitis B naïve by serology, ages 21 to 34, satisfied the inclusion and exclusion criteria, and their clinical labs were in the normal range or clinically insignificant (Table 2).

### Adverse events

One hundred thirty-one adverse events (AEs) occurred during the five-day post-vaccination period in 36 Senior volunteers; 112 were “mild,” 21 were “moderate”, and none were “serious”. Sixty reactions were local (redness, soreness) and 73 were systemic (malaise, fever, anorexia). Of the 133 AEs, 27 were judged to be “unrelated” to immunization, 125 (93%) resolved within 3 days of immunization and all resolved without sequelae. Five serious adverse events (SAEs) occurred over the 2-year follow-up period, and all were judged to be unrelated to immunization. Adverse events were not solicited in Junior volunteers.

### CT-visualized needle placement

The 1.0–1.5-in. (25.4–38.1 mm) vaccination needles penetrated well into the muscle in the 26 persons in the IM group. The depth of the deltoid fat pad determined by CT scan in the 26 IM Seniors measured before each of three vaccinations ranged from 5 mm to 21 mm, less than the tip of the 25.4–38.1 mm (1.0–1.5 in.) vaccination needles, which were inserted completely to the hub of the needle at right angles to the skin. The median depth of the deltoid fat pad was 9 mm. However, had a 5/8-in. (15.9 mm) needle been used, 3 (12%) of the IM group would have been vaccinated SC at least once, 2 persons vaccinated SC twice, and 1person vaccinated SC three times. Altogether, a 5/8-in. needle would have deposited vaccine into SC fat of 5 (10%) of the total cohort of 52 volunteers at least once.

### Serological responses

**Fig. 1:**
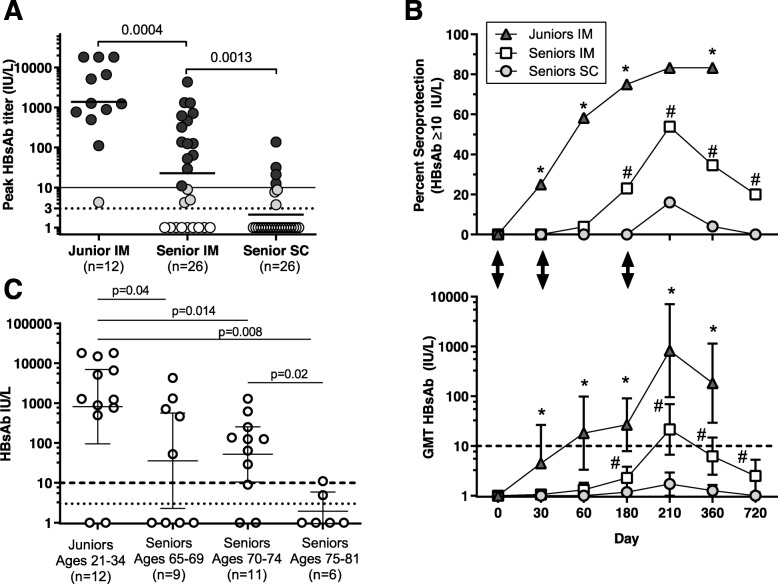
Anti-HBs serum Ab (HBsAb) in volunteers inoculated with Hepatitis B vaccine via IM (Juniors and Seniors) and SC (Seniors) routes. **a**. Peak antibody responses (GMT) are shown in each group. Seroconverters (> 3 IU/L) are shown as light gray filled circles, and seroprotected shown as solid circles. Significant differences between groups were determined by WMW tests. For statistical calculations of GMT, HBsAb values of < 3 IU/L are scored as 1 IU/L. **b**. Kinetics of HBsAb response to Hepatitis B vaccine in Junior and Senior volunteers inoculated via different routes. Upper panel: Percent of seroprotected volunteers (HBsAb ≥ 10 IU/L), with vaccination days indicated by arrows. Lower panel: GMT HBsAb over time. For statistical calculations of GMT, HBsAb values of < 3 IU/L are scored as 1 IU/L. Data include all specimens available for each time point: Juniors IM; *n* = 12, days 0–360. Seniors IM: *n* = 26, days 0–360; *n* = 25, day 720. Seniors SC: n = 26, days 0–180; n = 25, days 210 and 360; *n* = 22, day 720. The dashed line indicates the threshold for a protective response (10 IU/L). Significant differences by WMW test of area under the curve (AUC): *p* = 0.0007 Juniors IM vs. Seniors IM; *p* = 0.001 Seniors IM vs. Seniors SC. **c**. HBsAb titers at day 210 show reductions in seroresponses with increasing age. The heavy dashed line indicates the threshold for a protective response (10 IU/L); the light dashed line indicates the threshold for any response (≥ 3 IU/L). The geometric mean values and 95% confidence intervals for each group are shown. Significant differences between groups are shown (Wilcoxon-Mann-Whitney -WMW- test), nonsignificant differences are not shown. 10 of 12 Juniors (83%) achieved seroprotection at this timepoint, compared to 5 of 9 (55%) of Seniors ages 65–69, 8 of 11 (73%) Seniors ages 70–74, and 1 of 6 (17%) Seniors ages 75–81

Junior vaccine recipients were strongly seroresponsive, with 12 of 12 (100%) seroconverting (HBsAb ≥3 IU/L) and 11 of 12 (92%) achieving seroprotection (HBsAb ≥10 IU/L). Only 17 (65%) of 26 IM Seniors seroconverted (*p* = 0.038 compared to Juniors) and only 14 (54%) were seroprotected (*p* = 0.047 compared to Juniors). Of 26 SC Seniors, only 7 (27%) seroconverted and 4 (15%) were seroprotected. These rates were significantly lower than those observed in IM Seniors. Geometric mean titers (GMTs) of peak antibody levels at any time from Day 30 to Day 360 were significantly higher in Juniors compared to IM Seniors and in IM Seniors compared to SC Seniors (Fig. 1a). Further, the magnitude of response (peak HBsAb titer) was less for IM Seniors compared to Juniors (GMT 26 vs. 1389, *p* = 0.0004; Mann-Whitney-Wilcoxon -WMW- test). SC Seniors showed a further reduction in response magnitude, GMT 2.8 (*p* = 0.0013 compared to IM Seniors).

The temporal antibody response in Seniors was less robust than in the Juniors in terms of rapidity of appearance and levels of serum HBsAb achieved (Fig. 1b**, upper panel**). Specifically, a significantly higher proportion of Juniors compared to IM Seniors were seroprotected at every timepoint other than Day 210 (Fig. 1b**, upper panel**), and the GMTs were significantly higher in the Juniors at all time points as compared to IM Seniors (Fig. 1b**, lower panel**). The GMT was 39-fold higher in the Juniors than IM Seniors on Day 210. A significantly higher proportion of IM Seniors than SC Seniors were seroprotected as compared to SC Seniors. Likewise, GMTs were higher in IM Seniors on Days 180–720 (Fig. 1b). Finally, after the booster on day 180, there was a rapid decline in the percent of Senior IM vaccine recipients that remained seroprotected – from 54% at day 210 to 35 and 20% at days 360 and 720, respectively (Fig. 1b**, upper panel**).

The effect of age on hepatitis B vaccine-induced seroprotection was measured on Day 210, the day of expected peak immune response and 30 days after the final vaccine dose (Fig. 1c). A subgroup analysis of the IM Seniors showed a significant reduction in seroresponses even in the youngest subgroup (ages 65–69) compared to the junior cohort, with only 55% achieving seroprotection compared to 83% in the junior cohort. Among the eldest subgroup (ages 75–81), only 1 of 6 achieved seroprotection (17%). The junior cohort GMT was significantly higher than seniors of every age group, and significantly lower in the eldest seniors (ages 75–81) compared to those ages 70–74.

### Effect of gender on the immune response in IM seniors

**Fig. 2:**
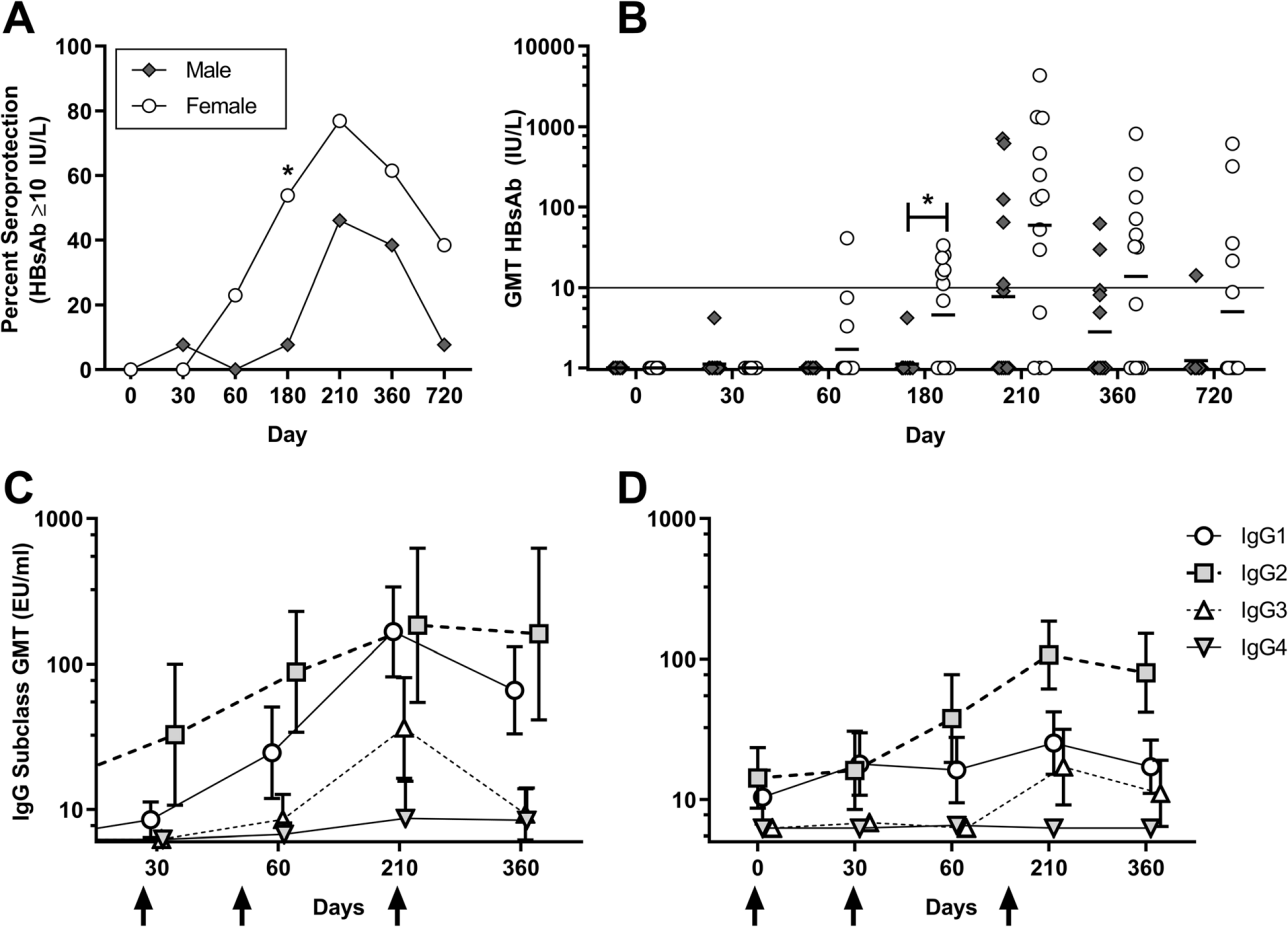
Kinetics of HBsAb titers for IM Seniors by gender and IgG subclass responses. **a**. Percent of individuals with titers above seroprotection threshold and **b**. Individual and geometric mean titers (horizontal lines) for men (shaded diamonds) and women (open circles) over time. The solid line indicates the threshold for protective response (10 IU/L). Significant differences by WMW test of AUCs: *p* = 0.084 Female vs. Male Senior GMTs. **c** and **d**. HBsAb IgG isotype profile in Juniors (*n* = 9) and Seniors (*n* = 16 days 0, 30, 60; *n* = 17 days 210, 360). Data represent GMT, with 95% CI indicated by error bars. Significant differences by WMW test of AUCs: *p* = 0.0019 IgG2 vs IgG1; *p* < 0.0001 IgG2 vs IgG3; *p* < 0.0001 IgG2 vs IgG4. IgG1 responses in Seniors were significantly lower than Juniors (*p* = 0.0007 by WMW test of AUCs)

In the IM Seniors group (13 women and 13 men), more women were seroprotected (≥ 10 IU/L) than men at all time points between Day 60 and Day 720, but the differences were significant only on Day 180 (*p* = 0.030); maximum protection occurred on Day 210 for both sexes (69% vs. 38%, *p* = 0.24) **(**Fig. 2a**)**. Similarly, GMTs were higher in women than in men at all time points between Day 60 and Day 720, although the differences were significant only on Day 180 (*p* = 0.008, WMW test). As measured by area under the curve (AUC) from Day 0 to Day 720, the response over time was stronger in women than in men (*p* = 0.048, WMW test), with women responding more quickly and with higher titers than men **(**Fig. 2b**)**. Of note, none of the women were taking systemic hormone replacement therapy, and there were no significant differences in the seroprotection rate or GMTs between the senior women who used Premarin (intravaginal estrogen, *n* = 5) and those who did not (*n* = 8). Analysis of SC responders was not done due to the small number of seroresponders (Fig. 1). In the Junior cohort, no significant differences were noted among the 6 male and 6 female volunteers, comparing the percent of volunteers seroprotected and AUC of GMTs across time points.

### Isotype distribution of IgG responses

Quantification of HBV-specific IgG subclass for each timepoint with detectable antibody titers demonstrated a consistent reduction across all classes in the senior cohorts (Fig. 2c-d). IgG2 isotypes were dominant in both junior and senior cohorts, followed by IgG1 (Fig. 2c). Both IgG2 and IgG1 responses peaked at day 210. The senior cohort displayed consistently blunted responses across all subclasses, significantly lower than juniors at days 210 and 360 for IgG1 isotypes. Additionally, while juniors generated broad responses including IgG2, IgG1, and IgG3 isotypes, the elderly IM group only mounted IgG2 responses above baseline.

### Effect of body fat and deltoid muscle fat on the immune response

We analyzed four measures of body fat in the Seniors: BMI, total body fat percentage by DEXA, percent fat in the vaccinated arm by DEXA, and the percentage fat in the vaccinated arm by CT. There was no statistically significant correlation between any of these four indices and the immune response, measured by the percentages of volunteers who seroresponded or who were seroprotected at any time from Day 30–360. Moreover, there was no correlation of the BMI and the individual HBsAb values on Day 210 (Spearman’s correlation). We did not measure body fat indices in the Juniors.

The percent fat in the deltoid muscle of the IM cohorts was similar comparing the 20 younger Seniors (65–74 years) and 6 older Seniors (75–81 years) (mean + SD of 6.8 + 2.8, and 7.2 + 2.6, respectively). The percentage total body fat or percentage fat in the vaccinated deltoid did not correlate with the percent achieving seroresponse or seroprotection (Student’s t-test) or GMT on day 210 (Spearman correlation). Similarly, the percentage fat in the deltoid muscle of the 13 IM male Seniors and 13 IM female Seniors was similar (mean + SD of 6.8 + 2.5 and 7.0 + 2.9, respectively), and there were no correlations by CT pixel analysis and DEXA with the immune responses measured by seroresponse, seroprotection, or GMT.

### T cell-mediated immune responses: proliferation

We also evaluated T cell-mediated responses induced by HBV vaccination. Proliferation of PBMC were measured following HBsAg stimulation. Juniors showed strong cumulative (days 30 to 360) proliferative responses since these were observed in 7 of 8 (88%) vaccinees. In contrast, only 12 of 45 (27%) Senior vaccine recipients exhibited these responses (Table 3). The route of immunization (IM vs. SC) in Seniors showed only minor differences in the percentages of volunteers proliferating (< 5% difference), but sex and age showed marked differences (≥10%). Higher percentages of females and 65–75 years old volunteers showed proliferation than males (33% vs. 20%, respectively) and volunteers over 75 years old (29% vs. 17%), respectively (Table 3).

**Table 3:** Percentage of individuals with HBsAg specific responses (proliferation) in Junior and Senior groups.

	Subsets	Proliferation		
		**Total**^**a**^	**Resp**^**b**^	**(%)**
**Juniors**		8	7	**88**
**Seniors**		45	12	**27**
**Seniors**	**IM**	25	6	24
	**SC**	21	6	29
**Seniors**	**Female**	24	8	**33**
	**Male**	20	4	**20**
**Seniors**	**65–74**	34	10	**29**
	**≥ 75**	12	2	**17**

^a^ Total evaluable volunteers at any time point during the study

^**b**^ Responders at any time point during the study

Responses for proliferation: ≥1.5 fold increases or ≥ 6000 cpm vs. day 0

Bolded % indicate a difference ≥ 10% among individuals in the compared groups

**Fig. 3:**
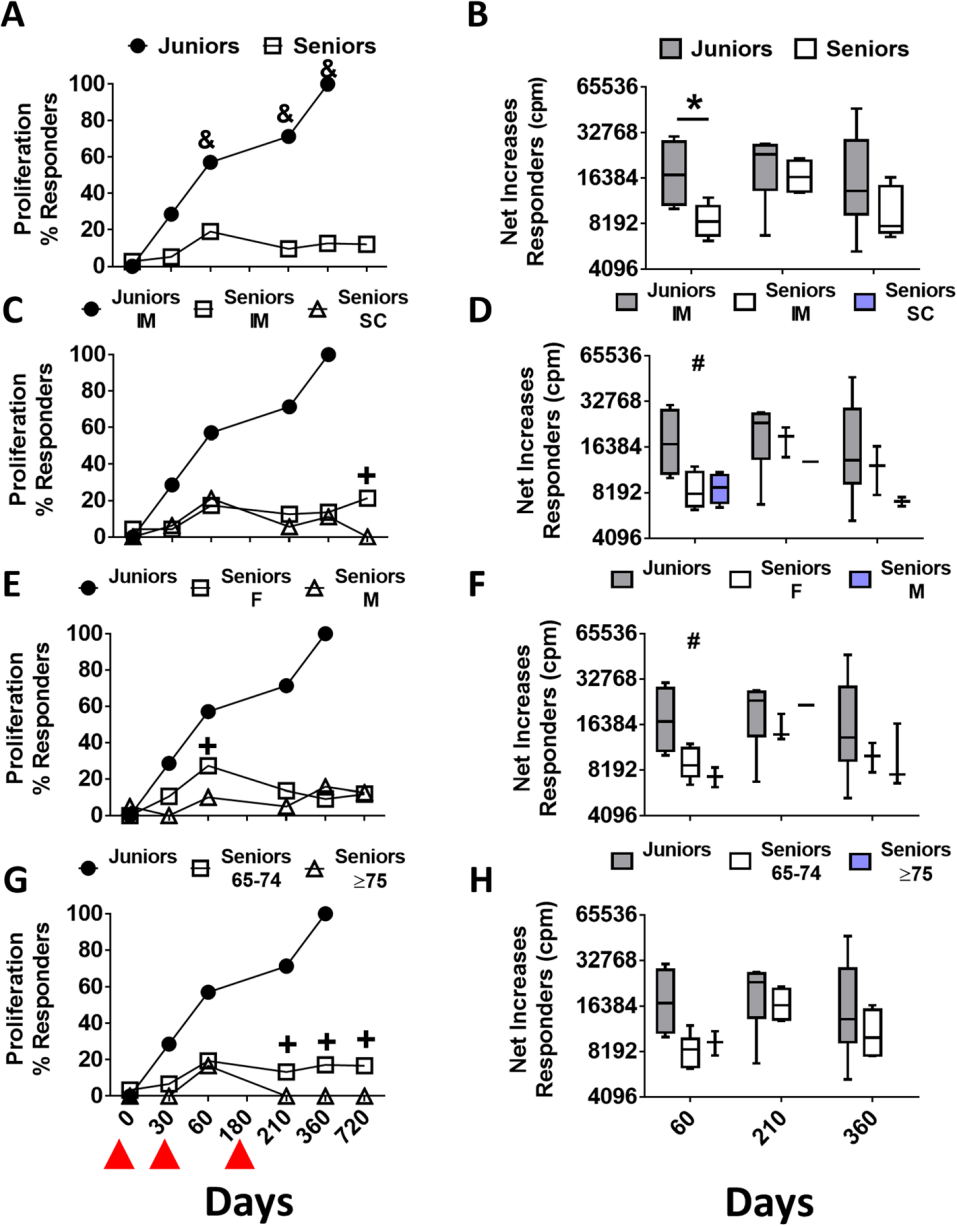
Kinetics of proliferative responses. Panel **a** shows the percentage of individuals in the Junior and Senior groups that showed proliferative responses at various time points upon stimulation with HBsAg at various time points. & indicate time points with ≥10% differences between Juniors and Seniors. Panel **b** shows the net proliferative responses (cpm) in Juniors and Seniors at 60, 210 and 360 days of the study (box and whisker plots). A WMW test was used to compare 2 groups (Seniors vs. Juniors). Significant differences (*p* < 0.05; *) between groups are indicated. Panels below show percent of vaccine recipients exhibiting proliferative responses by **c** route of vaccination (IM and SC), **e** sex (females and males), and **g** age subsets (65–74 and ≥ 75 years). + indicate time points with ≥10% differences between Senior subsets. Vaccination days are indicated with the Red Triangles. Panels **d**, **f** and **h** display the net proliferative responses (cpm) (days 60, 210 and 360) for Seniors split in subsets depending on route of vaccination, sex and age. Juniors and Seniors subsets at defined time points were compared using the Kruskal-Wallis test (#; *p* < 0.05) followed by the Dunn multiple comparisons procedure. Box and whisker plots display Min and Max values as well as the median. IM: Intramuscular SC: Subcutaneous. F: Females; M: males

Kinetics analyses showed higher percentages of Juniors with positive proliferative responses compared to Seniors at each time point after day 30 (> 10% differential) (Fig. 3a). Comparisons of net proliferative responses (cpm) were performed at days 60, 210 and 360, but only among individuals who showed proliferation. Juniors had enhanced proliferative responses at day 60, but not at days 210 and 360 (Fig. 3b). In subsequent analyses, Seniors were divided into subgroups depending on the route of immunization (IM or SC), sex and age. Differential responses were considered only after the second immunization. Some timepoints showed > 10% difference, but overall the responses among the Senior subgroups were comparable (Fig. 3c, e and g). Comparison of net proliferative responses (among responders) in the Senior subgroups were performed at days 60, 210 and 360 but no differences were identified. The Kruskal-Wallis test comparing Juniors and Senior subsets (e.g., Juniors IM, vs. Seniors IM vs. Seniors SC) showed significant differences between these groups depending on route of immunization and sex at day 60 (Fig. 3d and f, respectively). These results were expected since they reflect the differences identified in Fig. 3b. The other time points analyzed (days 210 and 360) showed no differences. In the subsets that involved age as subgrouping variable, no differences were identified at any of the time points analyzed (Fig. 3h).

### T cell-mediated responses: cytokine production

Typically, Th1 cells produce IFN-γ, IL-2 and TNF-α, while Th2 cells produce IL-4, IL-5, IL-6, IL-9, IL-10 and IL-13. To determine whether the immune responses induced by HBV vaccination involved Th1, Th2 or both we analyzed the patters of cytokine secretion of PBMC after HBsAg stimulation. IFN-γ, IL-2, TNF-α, IL-5, IL-4 and IL-10 were assayed. IL-10 was not detected at any of the time points; therefore, the results are not presented.

Among Juniors, strong rates of cytokine production were seen for IFN-γ (9 of 9; 100%), TNF-α (8 of 11; 73%), IL-5 (10 of 11; 91%), IL-4 (9 of 11; 82%), and IL-2 (9 of 11; 82%). In contrast, Senior responses were significantly lower: IFN-γ (29 of 47; 62%), TNF-α (15 of 50; 30%), IL-5 (17 of 50; 34%), IL-4 (17 of 50; 34%), and IL-2 (15 of 50, 30%) (Table 4). For every cytokine assayed, Juniors had > 10% more individuals than Seniors secreting these cytokines.

**Table 4:** Percentage of individuals with HBsAg specific responses (cytokine production) in Junior and Senior groups

	Subsets	IFN-γ			TNF-α				IL-5			IL-4				IL-2
		Total^a^	Resp^b^	(%)	Total^a^	Resp^b^	(%)	Total^a^	Resp^b^	(%)	Total^a^	Resp^b^	(%)	Total^a^	Resp^b^	(%)
Juniors		9	9	100	11	8	73	11	10	91	11	9	82	11	9	82
Seniors		47	29	62	50	15	30	50	17	34	50	17	34	50	15	30
Seniors	IM	25	18	72	26	7	27	26	7	27	26	7	27	26	6	23
	SC	21	11	52	23	8	35	23	10	43	23	10	43	23	9	39
Seniors	Female	25	16	64	27	10	37	27	8	30	27	8	30	27	9	33
	Male	21	13	62	23	6	26	23	9	39	23	9	39	23	6	26
Seniors	65–74	34	22	65	37	13	35	37	13	35	37	13	35	37	12	32
	≥ 75	12	7	58	12	2	17	12	4	33	12	4	33	12	3	25

^**a**^Total evaluable volunteers at any time point during the study

^**b**^Responders at any time point during the study

Responses for cytokines: ≥2 fold increases vs. day 0

Bolded % indicate a difference ≥ 10% among the compared groups

The Senior group was further analyzed by route of immunization, sex and age. A higher percentage of IM Seniors produced IFN-γ (≥10% differential) than SC Seniors, while > 10% more SC Seniors secreted IL-5, IL-4 and IL-2 than IM seniors. TNF-α production was comparable regardless of the route of immunization. Female Seniors showed a higher percentage of individuals producing TNF-α than males (37% vs. 26%). All other cytokines were generally comparable (< 10% differential) among Senior females and males. Finally, > 10% more Seniors under 75 years of age had enhanced TNF-α production compared to those over 75 years of age (35% vs 17%). Other cytokines were not affected by age (Table 4).

**Fig. 4:**
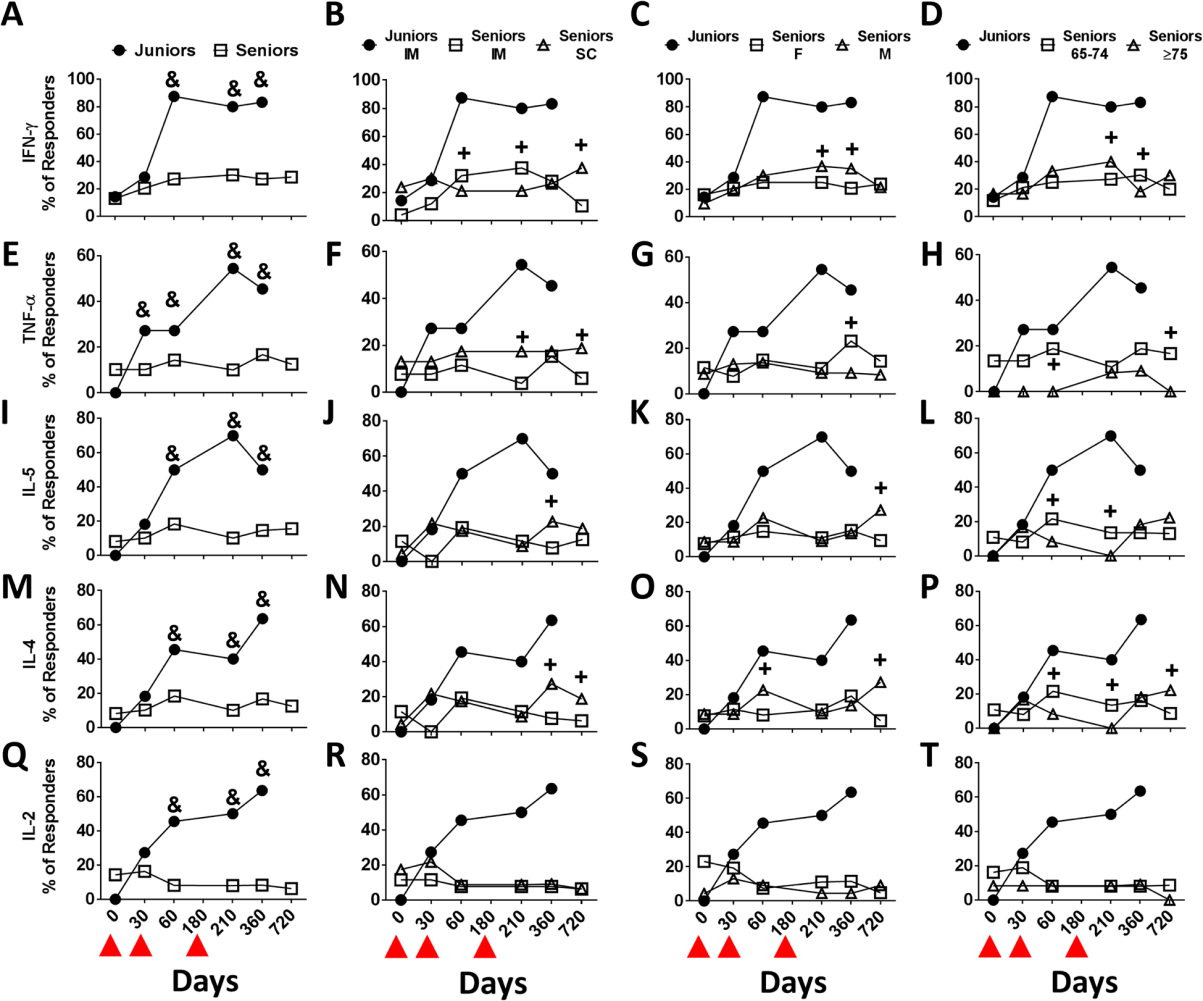
Kinetics of cytokine production. The leftmost panels show the percentage of individuals in the Junior and Senior groups that secreted cytokines after stimulation with HbSAg: **a** IFN-γ, (E) TNF-α, (I) IL-5, (M) IL-4, and (Q) IL-2 at various time points. (&) indicate time points with ≥10% differences between Juniors and Seniors after the second immunization. In subsequent panels in each row, the percentage of Seniors producing cytokines after stimulation was analyzed by the route of vaccination (IM and SC) **b, f, j, n** and **r**, sex (females and males) **c, g, k, o** and **s** and age subsets (65–74 and ≥ 75 years) **d, h, l, p** and **t**). Vaccination days are indicated with red triangles at the bottom of the Fig. IM: Intramuscular SC: Subcutaneous. F: Females; M: males. (+) indicate time points with ≥10% differences between Senior subsets after the second immunization

The kinetics of cytokine production showed that after the second immunization (>day 30), a higher percentage of Juniors (> 10%) produced cytokines than Seniors (Fig. 4a, e, i, m and q). Looking at the Senior subgroups (immunization route, sex, and age) there were some instances in which a > 10% difference in individuals producing cytokines were identified, but overall the responses were comparable (Fig. 4 f-h, j-l, n-p and r-t). In subsequent analyses we compared the net cytokines responses (ng/ml) between Juniors and Seniors at days 60, 210 and 360. These analyses were performed only among those individuals that showed positive cytokine responses. The capacity to produce IFN-γ and TNF-α among responders appeared comparable between Juniors and Seniors at all the time points. However, Juniors had a higher capacity to produce IL-5, IL-4 and IL-2 than Seniors at days 60 and 210 (Supplementary Fig. 1**, panels I, M, Q**). The Senior group was further analyzed depending on the immunization route, sex and age. Consistent with the proliferation analyses, no major differences were observed between the Senior subsets (Supplementary Fig. 1).

## Discussion

Prior studies have repeatedly demonstrated the poor response to vaccination in elderly cohorts, although SENIEUR-eligible individuals showed improved responses to influenza vaccines [34]. The SENIEUR participation criteria are very stringent, excluding ~ 90% of elderly individuals (requiring screening of 420 interested individuals to enroll 52 participants), but these criteria helped differentiate intrinsic age-related changes of the immune system from the changes which result from disease and medications [35]. In agreement with prior studies, we demonstrated lower seroprotection rates in healthy seniors compared to a junior cohort, delayed response to vaccination (with the majority of senior subjects achieving seroprotection only after the third dose), and lower peak titers. Furthermore, PBMC from healthy Seniors showed reduced HBsAg specific proliferation capacity and cytokine production (IFN-γ, TNF-α, IL-5, IL-4 and IL-2).

Inadvertent SC rather than IM vaccine deposition has been hypothesized to contribute to reduced immune responses. However, none of these studies carefully monitored whether the inoculum was placed in the subcutaneous tissue or in the muscle [36–38]. Herein we demonstrate reduced antibody responses of SC compared to IM delivery by directly visualizing by computed tomography the placement of needles into IM or SC vaccination sites, a major confounder of vaccination in elderly individuals. Notably, using 5/8-in. needles would have failed proper placement without visualization in 10% (5 of 52) of participants for at least one vaccination. It is likely that an improperly angled inoculum injection increases the placement of vaccine into SC fat. The elderly volunteers who received SC vaccinations had both significantly reduced rates of seroprotection and antibody titers. Interestingly, the proliferation capacity of PBMC does not appear to be affected by route of immunization. In fact, SC immunization resulted in increased production of IL-5, IL-4 and IL-2 (Table 4).

Similar to our study, a randomized controlled trial of quadrivalent, high dose influenza vaccine in Japanese adults over 65 years of age demonstrated higher seroconversion rates and geometric mean titers in participants receiving vaccine by the IM compared to the SC route [39]. For alum-adjuvanted subunit vaccines including hepatitis A and hepatitis B vaccines, where differences in immunogenicity were observed, IM administration has been consistently more immunogenic than SC [40, 41]. In children, SC hepatitis B vaccination appears to be efficacious with seroconversion rates comparable to IM [42]. Age-associated reductions in the influx of immune cells to lymph nodes may exaggerate the variations in tissue specific immune responses (e.g., IM vs. SC) between young and older populations [43].

The mechanism(s) behind differences in immunogenicity depending on the tissue in which the vaccine is deposited remain unclear. Studies have shown that SC administered immunoglobulin takes longer to be detected in plasma compared to IM administered immunoglobulin [44]. This suggests that the retention of antigens in the SC tissue due to reduced vascularity may result in poorer antigen presentation compared to IM injection, resulting in reduced immune responses when the vaccine is administered SC. However, it is also likely that variations in antigen trafficking and presentation from local injection sites to draining lymph nodes after vaccination may affect subsequent immune responses. Human muscle tissue contains few immune cells under normal conditions, but rapid recruitment occurs following inflammatory insults, which may be provided during vaccination by alum or other adjuvants for subunit vaccines, or directly by pathogen-associated molecular patterns of whole cell vaccines [45–47]. Additionally, there are differences in the cellular composition of muscle and skin tissues that might influence the outcome of vaccination. SC fat beds contain few immune cells; however, these are in close proximity to the skin dermal layers, which contain large numbers of lymphocytes, macrophages, and specialized dermal DCs that drain into local lymph nodes. On the other hand, muscle tissue contains few immune cells and very low DC numbers, but, as indicated above, these are rapidly recruited after an insult [48]. Studies in sheep have shown that the monocyte populations draining to lymph nodes is dependent on the site of inoculation, with CD16+ monocytes (associated with increased effector function) carrying the majority of antigen from IM inoculations, compared to SC inoculations for which there was a similar representation of CD16+ and CD16- monocytes [48, 49]. Taken together, differences in the presence of diverse immune cell subsets and physiological characteristics of SC and muscle tissues are likely to be responsible, at least in part, for the differences in the immune responses elicited by vaccination via the SC and IM routes. Unfortunately, mechanistic studies assessing the recruitment and mobilization of antigen presenting cells in humans is limited by the difficulties in obtaining skeletal muscle biopsies, while murine studies are limited by differences in macrophage and DC subsets between species as well as the physiological differences of fur-coated skin [45, 50]. Future experiments will be required to uncover the mechanism(s) underlying differences in immunity elicited by SC and IM vaccine administration in children, adults and the elderly.

Even when controlled for appropriate (IM) vaccine placement we demonstrate reduced seroprotection in an otherwise exceptionally healthy elderly population. Of importance, this reduction became more pronounced with advanced age. Among our cohort, seroprotection rates and antibody titers were higher in women than men, a difference that was sustained more than a year after vaccination. This reduced immune response was associated with reduced IgG1 levels in this elderly population. Use of Premarin was not associated with increased seroprotection or GMTs, although numbers were small. Interestingly, the percentage of fat within deltoid muscles was not associated with differences in response, nor was total body fat (DEXA) or BMI of the volunteer.

We also demonstrated marked reduction in T-CMI responses in both rate and magnitude of the overall response for Seniors compared to Juniors. Kinetic analysis showed that these differences between Juniors and Seniors were evident as early as after the second immunization. In Seniors, even responses in those who showed proliferation and cytokine production were reduced, which was more evident at day 60 for proliferation and days 60 and 210 for IL-5, IL-4 and IL-2 production. These results, which are generally in agreement with the results described in other systems, show that age affects either the kinetics (delayed responses) [51], the strength of the response, or both phenomena. Neither route of immunization nor gender in the Seniors appears to greatly influence the T-CMI responses, which was unexpected since both, route and gender, affected humoral responses (HBsAb titers) in this age group. Of note, advanced age appears to affect proliferation and cytokine production; however, the analysis found no statistically significant differences between cytokine production and proliferation, likely due to the small number of volunteers in this age group that exhibited responses at specific time points of the study. The significantly lower IgG1 response in IM Seniors compared to Juniors may reflect a particularly marked defect in Th1 responses in Seniors, as suggested by the reduced IFN-γ production by PBMC.

In our senior cohort, 10/24 (40%) male and 23/28 (85%) female volunteers were in the unhealthy (obese) range for percent body fat according to total body DEXA. Although obesity has been linked to perturbations in immunity, HBsAb responses were not associated with the amount of intramuscular fat or the total body fat in this study group. The absence of an association between HBsAb levels and intramuscular fat and total body fat, together with the absence of co-morbid risk factors of aging, refocuses attention on elucidating the intrinsic age-related defects of the immune system (i.e., immunesenescence) in otherwise robust seniors.

One limitation of this study is that, because the SENIEUR protocol is highly selective for an exceptionally healthy elderly population, it is not generalizable to the aging population at large. Additionally, our Senior cohort was inadvertently 94% Caucasian. Although this is unlikely to have had any effect, it is of note that our Junior and Senior cohorts received different lots of the commercially purchased vaccine due to the time lapse in enrollment. CMV seropositivity has been associated with limitations in vaccine-induced antibody responses [2, 52, 53], but immunity to CMV was not assessed in this study.

Some of the challenges of vaccination in the elderly population may be overcome by more potent adjuvants, such as the CpG-adjuvanted Heplisav-B which achieved seroprotection rates > 90% [13]. However, the molecular mechanisms that underly immunesenescence are complex and understanding of these mechanisms continues to evolve. Studies in exceptionally healthy Seniors will help to understand nonmodifiable senescence-associated immune changes, and to identify possible targets for enhancing immunization responses for elderly persons to achieve stronger, more durable, protective immunity.

## Conclusions

Vaccination of the elderly is complicated by reduced efficacy compared to younger populations. Divergent immune responses to vaccination in elderly individuals are often complicated by the comorbid or confounding conditions that accompany aging. Thus, we used a strict screening protocol (the SENIEUR Protocol) to select an exceptionally healthy elderly population in whom these confounders are absent or greatly minimized. We characterized the persistent immune dysfunction after vaccination in this population and provide actionable evidence to optimize vaccine efficacy. This is, to our knowledge, the first study that ensures that the vaccine inoculum is placed in the intended site (IM or SC) by visualizing the needle placement by computed tomography. We demonstrate reduced antibody responses of subcutaneous compared to intramuscular delivery as well as important differences after segregation by gender, as well as with advancing age. Th1 and Th2 T cell-mediated immunity were also altered in the elderly. A better understanding of this immune dysfunction is essential for improved vaccine design for the elderly. Importantly, these studies also provide practical solutions to improve vaccine responses, such as the use of longer needles to ensure proper vaccine placement, leading to improved disease prevention.

## Materials and methods

### Elderly (senior) cohort recruitment

A total of 613 community dwelling older volunteers age > 65 years were recruited from numerous sites in Baltimore. To study persons with inherent immunesenescence, elderly volunteers were screened and enrolled for their lack of co-morbid conditions that might confound interpretation of their immune response using a modified SENIEUR protocol (Table 1), resulting in the enrollment of 52 participants in the SENIEUR cohort [35].

### Young (junior) cohort recruitment

14 volunteers were recruited, 6 men and 8 women. The median age was 26 years old (range 21–34). All were Caucasian. Screening consisted of a medical history, targeted physical exam, and phlebotomy for complete blood count, thyroid stimulating hormone, serum vitamin B12, folate, and vitamin E, and a chemistry panel including AST/ALT, albumin, cholesterol, fasting glucose, BUN, creatinine, and hepatitis B serology. Exclusion criteria included active immunologically mediated diseases, immunodeficiency, severe cardiovascular disease, severe respiratory disease, malignancies, endocrine disorder, liver, renal, neural or gastrointestinal disease, malnutrition, active psychiatric disorder, drug or alcohol abuse, history of allergic reaction to Thimerosal, or pregnancy. Two of the 14 volunteers were excluded by positive serum HBsAb discovered at screening, leaving 12 volunteers in the Junior cohort. These volunteers were recruited at the Traveler’s Health Clinic or from the graduate schools at the University of Maryland. The demographic characteristics of the participants is shown in Table 2.

### The Recombivax-HB vaccine®

The recombinant HBsAg vaccine, an FDA licensed protein vaccine (Merck and Co., Inc., West Point, PA), was purchased commercially and administered as described below. A single lot of vaccine was used to immunize the 52 Senior volunteers. Because the Senior vaccine lot had expired, a second lot of Recombivax-HB vaccine was used to immunize the Junior cohort. The vaccine was stored in designated, locked storage refrigerators maintained at 4 °C - 8 °C and monitored by temperature alarms. Each group followed the identical three-dose vaccination schedule administered on days 0, 28, and 180.

### Vaccination guided by computed tomography (CT) scan of the arm

All elderly patients underwent computed tomography-guided vaccine injections. For junior volunteers, the distance measured from the acromial ridge to the injection site in the mid-deltoid region of each volunteer was recorded and used to position the injection site. The vaccine (10 μg; 1.0 ml) was injected into the deltoid muscle via a 1.0 or 1.5-in. needle and syringe, positioned at right angle to the skin over the deltoid muscle.

For elderly patients, a trained CT technician used a support to suspend the patient’s non-dominant shoulder and arm in midair. The patient’s arm was cleansed with alcohol, and a radiopaque marker was placed over the deltoid muscle held in place by tape. The distance measured from the acromion process to the marker was used for the first and the two subsequent CT scans (PQ 5000 CT scanner, Phillips Medical Systems, Bothell, WA) and vaccinations. Once properly positioned, a 1 cm thick slice of the deltoid was imaged, and the width of the subcutaneous fat pad and deltoid muscle was measured and recorded in cm using a scanning software (Supplementary Fig. 2). The patient was then randomized to receive either IM or SC immunization, as described below, and vaccinated on the CT table before his/her arm position had changed.

In the SC group, a 5/8 or 1-in. sterile needle attached to the vaccine syringe was measured to insure the needle tip was in the SC fat, at least 5 mm away from the deltoid muscle. The needle was inserted at a 90-degree angle to the surface plane of the skin position of the radioopaque marker. After injection, the needle was withdrawn without massage of the injection site to minimize forcing the vaccine inoculum from the SC space into muscle [25]. The single volunteer with < 6 mm of SC fat was not vaccinated. In the IM group, a 1.5-in. needle was measured and inserted 5 mm or more into the deltoid muscle. Two duplicate measurements were used to determine the needle insertion depth for all injections.

### Deltoid muscle fat content and density

Intramuscular fat content, as indirectly reflected by deltoid muscle density and the number of fat pixels within the muscle, was determined by standard CT image processing after each vaccination and was used as a covariate in the analysis. Computed Tomography images (PQ 5000 CT scanner, Phillips Medical Systems, Bothell, WA) acquired at the time of administration of vaccination were analyzed to determine mean Deltoid muscle density and to calculate the percentage of pixels containing fat. Images were first transferred to an open source image processing and analysis tool (ImageJ - http://rsb.info.nih.gov/ij/). Using the freehand drawing tool, three regions of interest (ROI) were independently drawn around the deltoid muscle on the site of vaccine administration (Supplementary Fig. 2). The numbers of pixels containing muscle and fat as well as mean muscle density, standard deviation and ROI area were recorded and subsequently used to calculate the percentage of fat content.

The percentage of fat in the deltoid muscle was determined using CT images, with slices taken at the site of injection. Images were taken using 120 or 130 kVP, 200–225 mAs, and 3.0, 8.0 or 10.mm slice thickness. A DICOM image viewer, ImageJ, was used to outline the region of interest (ROI), which was the deltoid muscle in each image. The software package also provided a histogram of pixel values in the ROI. Fat pixels were considered to be pixels with CT values in the range of − 10 to − 100 Hounsfield units (HU). Muscle Pixels were considered pixels in the range of + 14 to + 100 HU. All other pixels were considered outliers and were ignored in the calculation of fat percentage.

Percentage of fat in the muscle was determined by:

%fat = at Pixels / (Fat Pixels + Muscle Pixels) *100.

Total area (in mm^2^) of the ROI was calculated using the equation:

A_ROI_ = (Total Pixels) * (pixsize)^2^.

Here pixel size is in mm^2^ and Total Pixels refers to the number of Pixels in the ROI.

### Randomization by route of vaccination

After a subject had provide informed consent and had been determined to be eligible for the study, including CT imaging of the deltoid region showing the SC fat pad thickness to be ≥ 6 mm, the volunteer’s group assignment was made by the immunization nurse using a computer-generated randomization code. Only the immunization nurse knew the group assignment for the volunteer throughout the conduct of the study. She did not participate in evaluation of the study subjects. Although the volunteers were not told of their vaccination group, subjects could have inferred to which group they are assigned by visualizing the needle length used in their immunization. The investigators managing the blood specimens and conducting the immune assays were blinded to the route of vaccine administration.

### Follow-up of vaccinated volunteers

Local and systemic reactions were recorded for 5 days after each vaccination. Serum and peripheral blood mononuclear cells (PBMC) were isolated before the first vaccination (day 0) and on post-vaccination day 30 ± 2, 60 ± 4, 210 ± 7 days, and 360 ± 14 days for the Junior and Senior cohorts, and on day 720 ± 14 for the Seniors only. Serum was also collected at day 180 + 14 for antibody measurements.

### Determination of body composition

Body composition was assessed by Dual Energy X-ray Absorptiometry (DEXA) to determine composition of the deltoid muscle, total body fat and bone mass on all 52 members of the Senior cohort.

### Hepatitis B serology studies

Serum HBsAb titers were determined by a commercial laboratory (Quest Diagnostics, Baltimore, MD) using the Quantitative AUSAB test kit (Abbott Laboratories, Abbott Park, IL). In order to validate HBsAb results, 59 serum samples selected from 23 subjects initially assayed by Quest diagnostics were sent to a second commercial laboratory (ViroMED Commercial Laboratories, Minneapolis, MN). The sera were selected from volunteers vaccinated by the IM and SC routes, and from those with < 3 IU/L, 3–9 IU/L and ≥ 10 IU/L values. Agreement between both commercial labs was excellent. Only one of 59 serum specimens was non-concordant (11 IU/L by Quest, 9 IU/L by ViroMED). ViroMED provided quantitative titers on all serum samples with HBsAb of > 150 IU/L.

Anti-HBc (core) and anti-HBs (surface) antibody assay results were monitored for possible community-acquired HBV infection in the Senior cohort at Days 0, 180 and 360. Abbott commercial test kits were used (anti-HBc by the CORZYME-M test kit; and the HBsAg by the AUSZYME test kit (Abbott Laboratories, Abbott Park, IL).

### IgG subclasses

HBV specific IgG subclasses were measured by indirect ELISA in serum samples from individuals with HBsAb titers > 10 IU/L. ELISA plates were coated with 2 μg/ml of Recombivax antigen (Merck) for 2 h at 37 °C and blocked with 10% non-fat dry milk in PBS overnight at 4 °C. After each incubation, plates were washed with PBS-0.05% Tween 20 (PBST). Samples were tested in duplicate, in multiple 2-fold dilutions in 10% non-fat dry milk in PBST. IgG subclasses were revealed using biotin-conjugated murine monoclonal antibodies against human IgG1, IgG2, IgG4 and IgG4 (Hybridoma), followed by HRP-labeled avidin and TMB substrate. Titers were calculated from regression curves as the inverse of the serum dilution that produced an Absorbance value _450nm_ of 0.2 above the blank.

### Antigen-specific proliferative responses by PBMC

Cryopreserved PBMC were thawed and re-suspended in complete medium (RPMI 1640 containing 10% heat-inactivated human AB serum + 10 mM HEPES + 2 mM L-glutamine + 50 μg/ml gentamicin) and incubated at 37 °C, 5%CO_2_, in 96-well round bottom plates (2 × 10^5^ PBMC/well in triplicate) in the presence of various concentrations of HBsAg [0.01, 0.06 and 0.25 μg/ml]. Controls included stimulation of cells with media, BSA (0.01 and 0.06 μg/ml; negative controls) and plate-bound anti-CD3 plus anti-CD28 antibodies (positive controls). Six days after initiation of the assays, 1 μCi/well of ^3^H-TdR was added to each well and the cultures incubated for an additional 18 h. Cultures were terminated by automated harvesting and thymidine incorporation determined by liquid scintillation counting (counts per million; cpm). HBsAg-specific proliferative responses post-vaccination were defined as an increase ≥1.5 fold or ≥ 6000 cpm versus day 0, after background responses (BSA) at each time point were corrected (subtracted) in every individual.

### Antigen-specific cytokine production by PBMC

Cryopreserved PBMC were thawed, re-suspended in complete medium and incubated at 37 °C, 5% CO_2_, in 6-well plates (3 × 10^6^ PBMC/well in duplicate) and stimulated with various concentrations of HBsAg [0.12 and 0.25 μg/ml]. Controls included cells stimulated with BSA [0.25 μg/ml] (negative controls) and anti-CD3/CD28 coated plates (positive controls). Supernatants were harvested 3 days later and frozen (− 80 °C) until analysis. Interferon-γ (IFN-γ), tumor necrosis factor-α (TNF-α), interleukin (IL)-2, IL-4, IL-5 and IL-10 were assayed in the supernatants using Cytometric Bead Array (CBA) assay kits (Becton Dickinson, CA), following the manufacturer instructions. In short, cell culture supernatants were incubated with cytokine capture beads for 3 h. Standards for each cytokine evaluated were provided by the manufacturer and used in every experiment. The data were collected in a LSRII custom flow cytometry system. Approximately 3000 individual beads were collected for each supernatant. Cytokine levels (in pg/ml) in test samples were obtained by interpolation in curves generated using the recombinant cytokine standards. The levels of sensitivity were ~ 2.5–20 pg/ml depending on the cytokine evaluated. To minimize day-to-day variability, we evaluated all time points from each volunteer in a single assay. HBsAg-specific cytokine production by PBMC after vaccination was defined as an increase ≥2 fold (in ng/ml) versus day 0, after background responses (BSA) at each time point were corrected (subtracted) in every individual.

### Statistical analysis

HBsAb levels were summarized by percentage protected (HBsAb ≥10 IU/L) and geometric mean titer (GMT). Fisher’s exact test (FET) was used to compare immunological responses (percent HBs responders) between the two Senior vaccine groups (IM and SC), between the Junior IM and Senior IM groups, and between males and females in the Senior IM group. The Wilcoxon-Mann-Whitney (WMW) test was used for pairwise comparisons of GMTs among the three groups on any day after immunization; *p*-values were exact when there were no ties in ranks, and approximate, corrected for continuity, in case of ties. For calculation of GMT, HBsAb levels below the limit of quantitation (3 IU/L) were assigned the value 1 IU/L.

HBsAb levels over time were summarized within individuals by the area under the curve (AUC) [54]. For each individual, AUC was calculated for HBsAb levels, beginning at Day 0, as a sum of areas of rectangles determined by successive immune responses over time, where the width of the rectangle is the width in days of the interval between serum samples and the height is the mean of the immune responses at the beginning and end of the interval. AUCs were compared using the WMW test with continuity correction.

Proliferative responses and cytokine production levels in HBsAg stimulated PBMC among Juniors and Seniors (2 groups) were compared at specific time points (days 60, 210 and 380) using a WMW test. Comparison involving more than 2 groups (e.g., Juniors vs. Seniors males vs. Seniors females) used the Kruskal-Wallis test, followed by the Dunn’s multiple comparisons procedure for pairwise comparisons.

All statistical tests involving two groups were two-sided, and results with p-values < 0.05 were considered statistically significant. For the FET, the two-sided p-value was calculated as twice the smaller of the two one-sided p-values. No adjustment was made for multiple comparisons. Statistical analysis was done using NCSS (Number Cruncher Statistical Systems, Kaysville, Utah) and GraphPrism 6.0 for Windows (GraphPad, San Diego California USA).

## Supplementary information

**Additional file 1: Figure S1.** Cytokine production among responders. Panel A, E, I, M and Q show the net changes in cytokine production (ng/ml) among responders in Juniors and Seniors at 60, 210 and 360 days of the study (box and whisker plots). A WMW test was used to compare 2 groups (Seniors vs. Juniors); (*) indicates *p* < 0.05. A horizontal bar indicates the groups with significant differences. In the remaining panels in each row the Senior group was split by the route of vaccination (IM and SC) (B, F, J, N and R), sex (females and males) (C, G, K, O and S) and age subsets (65–74 and ≥ 75 years) (D, H, L, P and T). Junior and Senior subsets at specific time points were compared using the Kruskal-Wallis test, and the Dunn multiple comparisons procedure was used for pairwise comparisons after the Kruskal-Wallis test; (#) indicates p < 0.05. Horizontal bars within the subsets indicate those with significant (p < 0.05) responses. Box and whisker plots display Min and Max values as well as the median. IM: Intramuscular SC: Subcutaneous. F: Females; M: males. **Figure S2.** Computed Tomography image acquired at the time of administration of Hepatitis B vaccine. The image was analyzed to determine the width of the subcutaneous fat pad over the deltoid muscle from the radioopaque marker to the outer edge of the Deltoid muscle, and the width of the Deltoid muscle from the outer muscle edge to the bone. To obtain the mean Deltoid muscle density, a freehand drawing tool was used to outline three regions of interest (ROI) around the deltoid muscle at the site of vaccine administration (shown). Mean muscle density, standard deviation and ROI area in mm^2^ were determined in triplicate. Calculations shown for one representative patient at the first vaccination.

## Data Availability

The datasets used and/or analysed during the current study are available from the corresponding author on reasonable request by qualified investigators.

## Abbreviations

BMI: Body mass index
CBA: Cytometric Bead Array
CT: Computed tomography
ESRD: End-stage renal disease
HBsAb: Hepatitis B surface antibodies
HBV: Hepatitis B virus
HU: Hounsfield units
IFN-γ: Interferon-γ
IL-2: Interleukin 2
IM: Intramuscular
PBMC: Peripheral blood mononuclear cells
rHBsAg: Recombinant HBV Surface Antigen
ROI: Regions of interest
SAEs: Serious adverse events
SC: Subcutaneous inoculation
T-CMI: T cell-mediated immune responses
TLRs: Toll-like receptors
TNF-α: Tumor necrosis factor-α
