# Supplementary material for: The SENIEUR protocol and the efficacy of hepatitis B vaccination in healthy elderly persons by age, gender, and vaccine route
Source: Immun Ageing. 2020 Apr 28;17:9. doi: 10.1186/s12979-020-00179-9 (PMC7187507; doi:10.1186/s12979-020-00179-9)
Supplement: Supplementary file 1 — Additional file 1: Figure S1. Cytokine production among responders. Panel A, E, I, M and Q show the net changes in cytokine production (ng/ml) among responders in Juniors and Seniors at 60, 210 and 360 days of the study (box and whisker plots). A WMW test was used to compare 2 groups (Seniors vs. Juniors); (*) indicates p < 0.05. A horizontal bar indicates the groups with significant differences. In the remaining panels in each row the Senior group was split by the route of vaccination (IM and SC) (B, F, J, N and R), sex (females and males) (C, G, K, O and S) and age subsets (65–74 and ≥ 75 years) (D, H, L, P and T). Junior and Senior subsets at specific time points were compared using the Kruskal-Wallis test, and the Dunn multiple comparisons procedure was used for pairwise comparisons after the Kruskal-Wallis test; (#) indicates p < 0.05. Horizontal bars within the subsets indicate those with significant (p < 0.05) responses. Box and whisker plots display Min and Max values as well as the median. IM: Intramuscular SC: Subcutaneous. F: Females; M: males. Figure S2. Computed Tomography image acquired at the time of administration of Hepatitis B vaccine. The image was analyzed to determine the width of the subcutaneous fat pad over the deltoid muscle from the radioopaque marker to the outer edge of the Deltoid muscle, and the width of the Deltoid muscle from the outer muscle edge to the bone. To obtain the mean Deltoid muscle density, a freehand drawing tool was used to outline three regions of interest (ROI) around the deltoid muscle at the site of vaccine administration (shown). Mean muscle density, standard deviation and ROI area in mm2 were determined in triplicate. Calculations shown for one representative patient at the first vaccination. [file 12979_2020_179_MOESM1_ESM.pptx]

## Slide 1
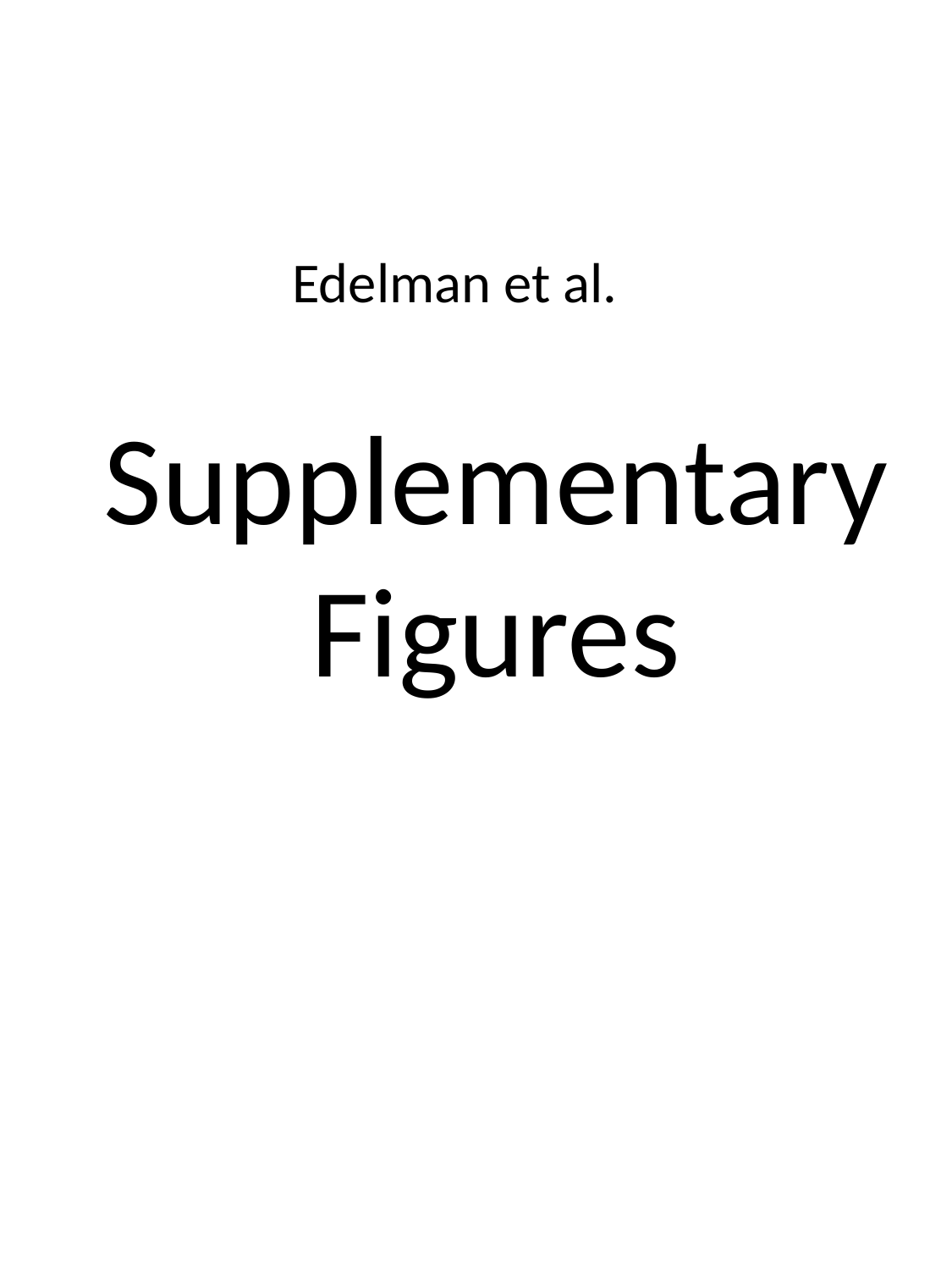

Edelman et al.
Supplementary Figures

## Slide 2
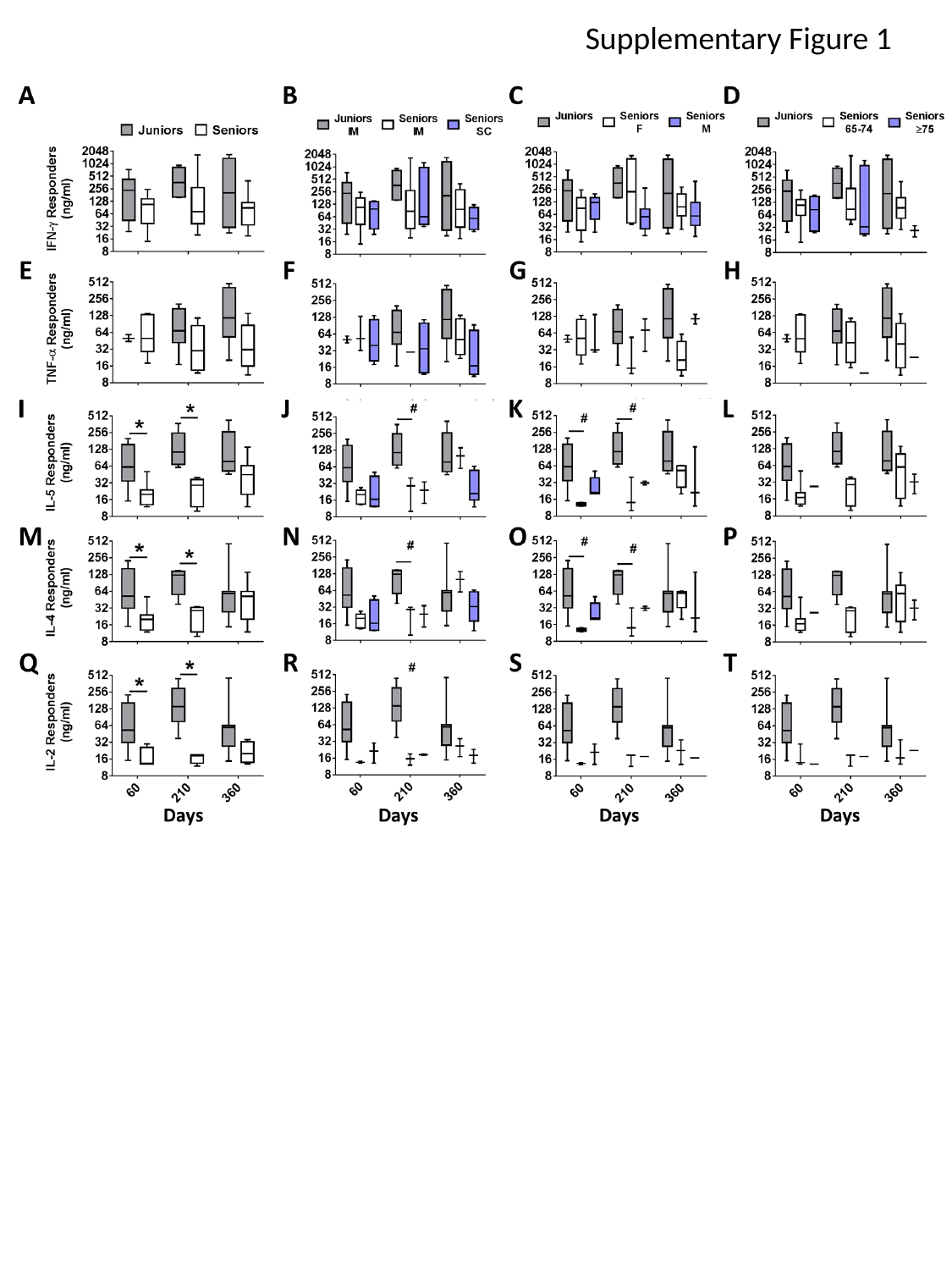

Supplementary Figure 1

## Slide 3
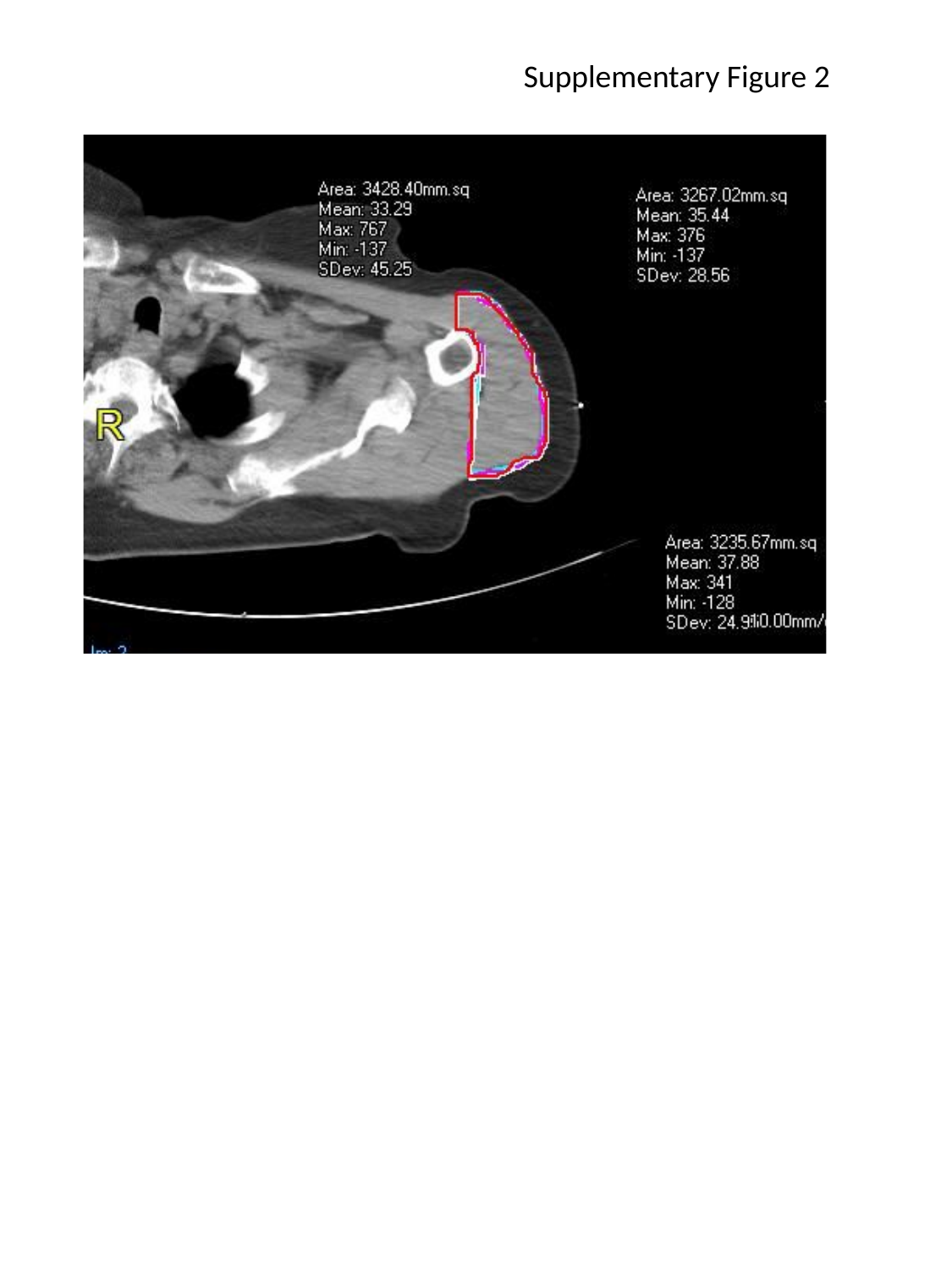

Supplementary Figure 2
